# Preparing for the future offspring: European perch (*Perca fluviatilis*) biosynthesis of physiologically required fatty acids for the gonads happens already in the autumn

**DOI:** 10.1007/s00442-023-05480-0

**Published:** 2023-11-17

**Authors:** Cyril Rigaud, Kimmo K. Kahilainen, Marco L. Calderini, Matthias Pilecky, Martin J. Kainz, Marja Tiirola, Sami J. Taipale

**Affiliations:** 1 Department of Biological and Environmental Sciences, University of Jyväskylä, Jyväskylä, Finland; 2 Lammi Biological Station, University of Helsinki, Lammi, Finland; 3 WasserCluster Lunz–Inter-university Center for Aquatic Ecosystem Studies, Lunz am See, Austria; 4 Danube University Krems, Research Lab of Aquatic Ecosystem Research and Health, Krems, Austria

**Keywords:** DHA, ARA, fads2, Perch, PUFA

## Abstract

Long-chain polyunsaturated fatty acids (PUFA) are critical for reproduction and thermal adaptation. Year-round variability in the expression of *fads2* (fatty acid desaturase 2) in the liver of European perch (*Perca fluviatilis*) in a boreal lake was tested in relation to individual variation in size, sex, and maturity, together with stable isotopes values as well as fatty acids (FA) content in different tissues and prey items. ARA and DHA primary production was restricted to the summer months, however, perch required larger amounts of these PUFA during winter, as their ARA and DHA muscle content was higher compared to summer. The expression of *fads2* in perch liver increased during winter and was higher in mature females. Mature females stored DHA in their gonads already in late summer and autumn, long before the upcoming spring spawning period in May. Lower δ^13^C_DHA_ values in the gonads in September suggest that these females actively synthesized DHA as part of this reproductive investment. Lower δ^13^C_ARA_ values in the liver of all individuals during winter suggest that perch were synthesizing essential FA to help cope with over-wintering conditions. Perch seem able to modulate its biosynthesis of physiologically required PUFA in situations of stress (fasting or cold temperatures) or in situations of high energetic demand (gonadal development). Biosynthesis of physiologically required PUFA may be an important part of survival and reproduction in aquatic food webs with long cold periods.

**Supplementary Information:**

The online version contains supplementary material available at 10.1007/s00442-023-05480-0.

## Introduction

At high latitudes, the winter lasts longer than the growing season and has a disproportionally large impact on organisms’ growth, reproduction, survival, and fitness (Studd et al. 2021; Sutton et al. 2021). Winter is a brutal annual selection that filters out genotypes and phenotypes unable to cope with its challenges (Studd et al. 2021). Consequently, winter has shaped survival strategies of many species, which in response developed various behavioral and/or physiological adaptations: migration (winter avoidance), hibernation, denning, communal housing, growth of a winter pelage, pre-winter fattening (i.e., hyperphagia), as well as mechanisms of energy savings such as protein sparing, among others (Hissa et al. 1998; Mustonen and Nieminen 2018; Pond et al. 1994; Shimozuru et al. 2016; Thouzeau et al. 1997). However, despite putting exceptional constraints on wild organisms, winter remains less studied than other seasons (Studd et al. 2021).

Many aquatic and terrestrial animals store lipids well ahead of winter to cover over-wintering and reproduction costs (Fernandes and McMeans 2019; Hissa et al. 1998; Mustonen and Nieminen 2018; Shimozuru et al. 2016; Thiemann et al. 2006). In fish, gonadal development comes at very high energetic costs and likely requires a high-quality diet (Jobling et al. 1998). Long-chain polyunsaturated fatty acids (LC-PUFA) such as arachidonic acid (ARA, 20:4ω6), eicosapentaenoic acid (EPA, 20:5ω3), or docosahexaenoic acid (DHA, 22:6ω3) are required for reproduction (e.g., for eggs and subsequent embryo development), neuronal development and membrane fluidity (thermal adaptation), among other processes (Pilecky et al. 2021; Taipale et al. 2018; Tocher 2003; Xu et al. 2022). Consequently, these LC-PUFA are critical for freshwater fish during over-wintering and reproduction. Because of the need to provide these physiologically required PUFA in adequate quantities to the embryos before they start feeding themselves, the combination of spawning and overwintering in freshwater fish likely imposes a greater energetic load on females than on males (Jobling et al. 1998).

Aquatic ecosystems occupy a unique position on earth as the main dietary source of n-3 LC-PUFA for all animals, including consumers of terrestrial ecosystems (Gladyshev et al. 2009). Herbivorous terrestrial vertebrates lack dietary LC-PUFA, but have easy access to their essential precursors (linoleic acid, LA, 18:2ω6, and alpha-linoleic acid, ALA, 18:3ω3) via terrestrial plants, and are able to convert them to LC-PUFA (Geiser et al. 2007; Kouba and Mourot 2011; Wood et al. 2008). However, most of these studies are limited to lab-raised, domesticated, or hatchery-reared animals (Twining et al. 2016). In aquatic ecosystems, only a few taxa of phytoplankton (e.g., cryptophytes, chrysophytes, diatoms, and dinoflagellates) are responsible for most of the EPA/DHA synthetized globally (Taipale et al. 2016). The occurrence of these algal groups in freshwater lakes is influenced by multiple physico-chemical parameters (Reynolds 1992). From a seasonal perspective, the open water season is a fast-growing period for juvenile fish, but also the best period to store lipids and other nutrients for most aquatic consumers. Current ongoing global environmental change may reduce the LC-PUFA content in the seston of lakes and thus their transfer to the pelagic food web (Kesti et al. 2022; Taipale et al. 2019). This may have deleterious consequences on growth, survival and reproduction of aquatic consumers (Taipale et al. 2022). The consumers usually need to receive LC-PUFA directly from their diet (e.g., typically from aquatic sources) or alternatively have the ability of bioconversion from precursors.

Even though fish usually live in PUFA-rich environments, they may need to biosynthesize physiologically required PUFA, such as ARA, EPA, and DHA. This synthesis from the essential C18 precursors (LA and ALA) involves a series of desaturation and elongation reactions catalyzed by fatty acid desaturase (Fads) and elongation of very long-chain fatty acid (Elovl) enzymes (reviewed in Xie et al. 2021), respectively. The *fads2* gene, encoding for the fatty acid desaturase responsible for some of the desaturation steps mentioned above, is widely present in teleost fish. Both *fads1* and *fads2* are present in mammals, reptiles and birds, and show a lineage-specific expansion of the *fads1* gene in the latter two groups (Castro et al. 2012). Because of its key rate-limiting role in the biosynthesis of physiologically required PUFA in fish (Bláhová et al. 2020), research regarding *fads2* in teleost has gained traction over the last decade, partly motivated by the goal of increasing the nutritional value of farmed fish for human consumption (Monroig et al. 2013; Xie et al. 2022). However, few studies focused on ecological endpoints. While some authors have explored the variability of the enzymatic activity of Fads2 between species with different foraging and ecological traits (Xie et al. 2020), there is, to our knowledge, no study regarding its seasonal variability. Gaining knowledge on the subject would help understand if and how freshwater fish species and other animals are able to modulate their endogenous ARA, EPA, or DHA production based on physiological needs (e.g., investment in reproduction) or to help survive in situations of deprivation of these molecules (e.g., during over-wintering).

The European perch (*Perca fluviatilis*) is a cool water species with an optimal temperature of 23 °C (Fiogbé and Kestemont 2003) and one of the most common fish species in Europe, thriving in lakes, ponds, and rivers. Its growing season varies based on latitude, but in boreal regions it is limited to the summer months (June to August). Perch can tolerate a large variety of environmental conditions, has an effective reproduction, and thus can reach high population densities in optimal conditions. Perch may grow up to 60 cm in length and typically shows ontogenetic diet shifts, first from zooplankton to benthic macroinvertebrates and later to feeding on fish, including commonly cannibalism (e.g., Svanbäck et al. 2015). However, individual and population level variation in perch diet is wide (Sánchez-Hernández et al. 2021). The reproductive investment in gonads is significantly higher in females compared to males, which suggests potential differences in fatty acid content, as well as requirements to biosynthesize physiologically required fatty acids to some extent. The *fads2* gene was recently cloned and partially characterized in *P. fluviatilis*, where it displayed a functional ∆6 desaturation activity towards C18 precursors, as well as high expression in the liver, brain, and intestine compared to other tissues (Geay et al. 2016). While Geay et al. (2016) were not able to test or demonstrate ∆5 and ∆4 desaturation activities towards C20 and C22 precursors (respectively), they described two alternative splicing transcripts of *fads2* that were also highly expressed in the liver and brain of perch. These authors hypothesized that these alternative transcripts may be responsible for the ∆5 and ∆4 desaturation steps mentioned above. Alternative *fads2* splicing transcripts are common in fish (González-Rovira et al. 2009; Matsushita et al. 2023; Santigosa et al. 2011), likely to compensate for the loss of the *fads1* gene. Moreover, additional experimental evidence in perch fed either low or high LC-PUFA diets strongly suggest its ability to biosynthesize DHA (Geay et al. 2015). Yet, it is unknown if *fads2* expression in the perch varies seasonally and/or depending on sex and maturity.

In the present study, we investigated the year-round variability in the expression of *fads2* (measured via quantitative real-time PCR) in the liver of the European perch in a boreal lake in relation to individual variation in size, sex, and maturity. Our objectives were to assess; (i) seasonal changes in the expression of *fads2* in the liver of *P. fluviatilis*, and; (ii) how such changes were related to environmental (seasonality in temperature, light, oxygen) or biological variation (body size, sex, maturity, diet). Together with *fads2* activity we report values of carbon stable isotopes as well as the content of fatty acids (FA) in perch tissues (liver, muscle and gonad) and potential perch prey items that were sampled simultaneously.

## Materials and methods

### Sampling site and samples collection

The study site, Lake Pääjärvi, is a humic and mesotrophic boreal lake located in southern Finland (61°04ʹ N, 25°08° E) (Supplementary Table S1). It has a surface area of 13.5 km^2^, an average depth of 14.4 m (maximum depth of 85 m), and a shoreline length of 33 km (Piro et al. 2023; Ruuhijärvi 1974). Water physical and chemical parameters in Lake Pääjärvi during the sampling period are presented in the Supplementary Materials (Supplementary Table S1). While some parameters such as oxygen saturation, nutrients, or total carbon levels were quite stable all year round, other parameters such as temperature or chlorophyll-a concentration (depth 0–2 m) showed pronounced seasonal variation, peaking in summer and reaching minimum during mid-winter, as expected in a system such as a boreal lake. February was the coldest month included in our sampling and was characterized by the presence of a thick ice cover (Supplementary Table S1).

Seston was collected from October 2020 to December 2021 for FA analyses from the deepest part of lake at the surface (0–1 m depth) by sieving 2 L of water through 50 µm mesh size and then filtering it through GF/C filter paper. Filter papers were immediately stored at − 80 °C. Zooplankton samples were taken from the same location from August 2020 to September 2021 using vertical tows of 0–80 m with 1 m diameter and 3 m long plankton net with a mesh size of 500 µm. Animals were sorted in the laboratory to Calanoida and *Mysis relicta*, those were separately stored in 2 mL plastic vials and frozen at − 80 °C.

Perch were captured every two to three months from September 2020 to August 2021 in the littoral habitat, using a gillnet series of 1.8 m high × 30 m long with mesh sizes ranging from 5 to 60 mm. Lake was ice-covered from the beginning of January to the beginning of April 2021. The total length (accuracy 1 mm) and mass (accuracy 0.1 g) of each perch were measured. Fish were selected to cover different size categories and both sexes, except in February 2021 when total sampling was limited. Length categories were split into small perch (< 12 cm), medium perch (12–20 cm), and large perch (> 20 cm). Fulton’s condition factor was calculated for each perch: CF = (*M*/TL^3^) × 100, where *M* is the fish mass in grams, and TL is the total fish length in cm. For the perch captured between September 2020 and April 2021, a piece of fresh liver tissue of each fish was collected and immediately homogenized in 750 μL of DNA/RNA Shield using a TerraLyzer™ Cell Disruptor and BashingBead™ (2.0 mm) lysis tubes (Zymo Research, Irvine, CA, USA), for later gene expression analyses. A second piece of liver tissue was sampled and kept separately for FA analyses. White dorsal muscle tissue was sampled for the same purpose, next to the anterior dorsal fin and above the lateral line of each fish. Samples were stored at − 80 °C, while the remaining carcasses of each fish were stored at − 20 °C. Later, fish were thawed, and stomach content was analyzed using the points method (Hynes 1950), where stomach fullness was scored from 0 (empty) to 10 (full), and the volumetrical proportion of each prey category was visually determined. Sex and sexual maturity were assessed visually from gonads, and gonadosomatic index (GSI) was calculated as GSI = GM/SM × 100, where GM is the gonad mass, and SM is the somatic mass. Finally, gonad tissue was also sampled for FA analyses.

### *fads2* gene expression in the liver

Detailed methods are available in the supplementary materials. Briefly, RNA was extracted from liver samples using a Chemagic™ 360 and the Chemagic™ Viral DNA/RNA 300 Kit. The RNA was treated with DNAse and reverse transcribed to cDNA. cDNA samples were stored at − 20 °C. The primer sequences for *fads2*, *ef1-α*, and *β-actin* were obtained from the literature (Geay et al. 2016). All amplification reactions were done in a volume of 25 μL and run on a CFX96 Real-Time PCR cycler. A single melting temperature peak was observed in the dissociation curves for each pair of primers and no amplification was observed in the negative controls. For each sample, the *fads2* gene expression (efficiency corrected) was calculated using the CFX Maestro™ software.

### Fatty acids content and stable isotopes analysis

Detailed methods are available in the supplementary materials. Total lipids were extracted from seston, zooplankton, and fish tissues using the protocol described by Folch et al. (1957). Analyses were performed using a gas chromatograph (Shimadzu) equipped with a mass detector (GC–MS) and using helium as a carrier gas and an Agilent DB-23 column. The identification and quantification followed a previously published method (Taipale et al. 2016). The fatty acid FAME (Larodan AB) was used as the internal standard for the correction of sample FA concentrations.

The δ^13^C values of FAs were determined using a GC-MSD (Agilent 7890B GC, Agilent 5977B MSD) connected to an Isotope Ratio Mass Spectrometer (Isoprime precisION, Elementar) via combustion interface. Fatty acids were separated using a 30 m ZB-23 column (Phenomenex). The samples were run against an internal standard, FAME. The δ^13^C values of FAME were run with EA-SIRMS system (Thermo Fisher Scientific), and were used to calculate the δ^13^C value of individual FA.

### Data and statistical analyses

The normal distribution of the datasets was tested with the Shapiro–Wilk test. As most datasets were not normally distributed, differences among the groups of individuals or time points were investigated using non-parametric Kruskal–Wallis tests (KW). When significant differences were found, it was followed by a Dunn's post hoc test. For most comparisons, individuals were grouped by sampling months, sex and maturity stages (mature females, immature females, mature males, immature males, and juveniles). Correlations between the *fads2* gene expression in the perch liver and the FA content (ARA or DHA) in the perch tissues were assessed using the Pearson’s correlation coefficient (*r*) test.

Enzymatic processes usually use ^12^C faster than ^13^C (Twining et al. 2020). Isotopically lower δ^13^C values can be expected when a molecule is synthesized from a precursor rather than taken directly from the diet or mobilized from other tissues. For the comparison of the δ^13^C values of the FA in the perch tissues versus the prey items, individuals were separated by size: small (< 12 cm), medium (12–20 cm), and large (> 20 cm). Due to strong female-biased sexual dimorphism in perch (Estlander et al. 2017), only a few large males were collected, thus the latter category (> 20 cm) was restricted to large, mature females. Large perch are known to exhibit cannibalism by preying on younger, smaller perch, as observed frequently in our study lake (Laiho 2022). As no other fish species were sampled in the present study, the δ^13^C values of the FA in the muscle tissue of small perch (< 12 cm) were used as a proxy of potential fish prey items for the larger individuals. The δ^13^C values obtained from the seston and pooled zooplankton samples (Calanoida and *Mysis relicta*) were grouped and plotted by season (summer and winter) since, for several sampling months, only one sample was collected. Summer included months from May to September and winter from October to April. To be coherent for all prey items, the δ^13^C values in the muscle tissue of small perch were grouped the same way when used as a proxy of fish prey for large perch.

All statistical analyses were done using R 4.2.0 (The R Foundation for Statistical Computing) with the significant level set at *α* = 0.05.

## Results

### Perch life history parameters and diet

Mature females were the largest individuals captured all year long (Supplementary Table S2). The gonadosomatic index (GSI) of mature females increased gradually between September and its peak in April (before spawning in May), then decreasing to the lowest level in the summer months (June and August) (Supplementary Table S2). Mature males, on the other hand, had their highest GSI in September (higher compared to April, June, and August 2021; KW, *p* ≤ 0.05). It then declined slightly during the winter months while remaining relatively stable until the spawning period, before being at its lowest during summer, as observed with mature females (Supplementary Table S2). Juveniles were only available in February (*N = *2) and were the smallest individuals captured.

For all individuals, the percentage of recorded empty stomachs was at its highest in September, February and April and close to 0% for the other sampling months (December, June, and August). Accordingly, the average stomach fullness index score was higher in December, June, and August compared to September, February, and April (KW, *p* ≤ 0.05) (Supplementary Table S2). Analysis of stomach content revealed that *Mysis relicta* was an important part of the diet for both small (< 12 cm) and medium-sized (12–20 cm) perch during the winter months (December, February, and partially in April) (Supplementary Table S3). During spring and summer, small perch preyed mostly on small zooplankton (Copepods from the order Cyclopoida and Calanoida). The summer diet of medium-sized perch was more diverse and included small zooplankton, insects, as well as other fish (Supplementary Table S3). Large individuals were almost exclusively piscivorous all year long, especially large females. Smaller perch were an important diet item for larger piscivorous perch, representing up to 60–70% of the observed diet in February and August, for example (Supplementary Table S3).

### Polyunsaturated fatty acids content in the food chain

**Fig. 1:**
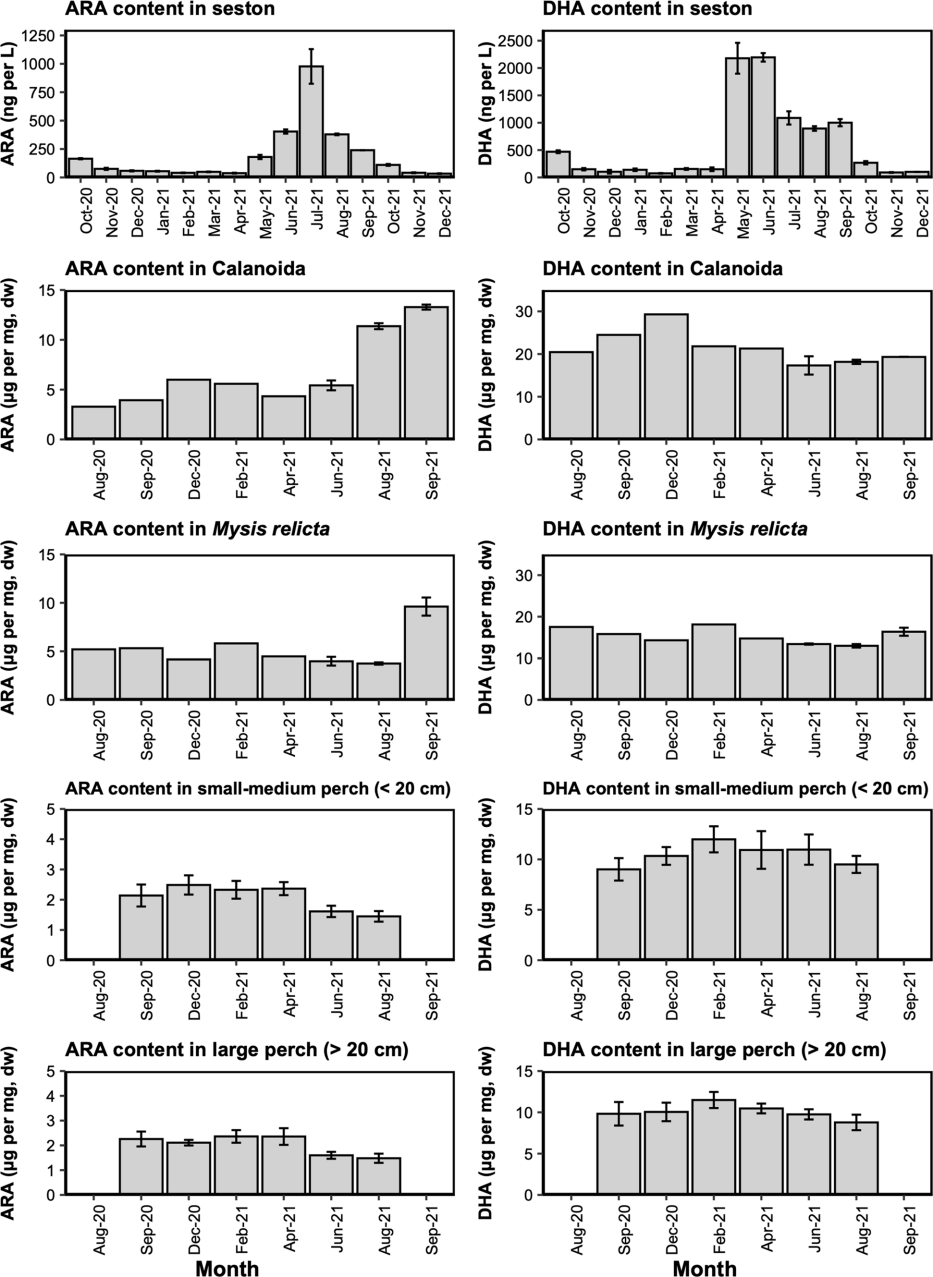
Average concentrations of ARA (left plots) and DHA (right plots) in the seston (*N = *2, ng per L of lake water), in the perch prey items Calanoida and *Mysis relicta* (*N = *1–2, μg per mg of tissue, dry weight, pooled individuals) and in the perch muscle (*N = *14–20, μg per mg of tissue, dry weight) sampled in Lake Pääjärvi between 2020 and 2021

PUFA content was measured in the seston and in some prey items of perch in Lake Pääjärvi, *Mysis relicta*, and Calanoida, as well as smaller perch which are being predated by larger ones (Fig. 1). Seston ARA and DHA content showed large seasonal variations, with 24- and 29-fold differences between the lowest values in February and the peak during spring/summer, respectively. The DHA content in seston was quite low from October to April, then peaked in May–June and decreased slightly in the following months until reaching low values again in autumn. ARA exhibited a similar trend with low values in autumn and winter, however, its peak occurred later (July), and the increase was more gradual in spring compared to DHA. Calanoida had slightly higher DHA content in December, while a large peak was observed for ARA during the summer of 2021 following a seston ARA peak (Fig. 1). The ARA and DHA content of *Mysis relicta* appeared relatively stable all year round, except for ARA peaking in late summer 2021, as observed with Calanoida (Fig. 1).

The ARA and DHA content in perch muscle followed similar trends between small-medium invertivorous perch and larger piscivorous perch (Fig. 1). The ARA content was significantly (KW, *p* ≤ 0.05) lower during the summer (June and August) compared to the rest of the year, with maximum values ranging between 2 and 3 μg per mg. Similarly, the DHA content in perch muscle was lowest in August and September, but peaked during winter: it was higher in February compared to any other month (KW, *p* ≤ 0.05). Interestingly, juveniles that were sampled in February (*N = *2) had the highest DHA content (~ 14 μg per mg) compared to larger individuals.

### Tissue-specific PUFA patterns in perch

**Fig. 2:**
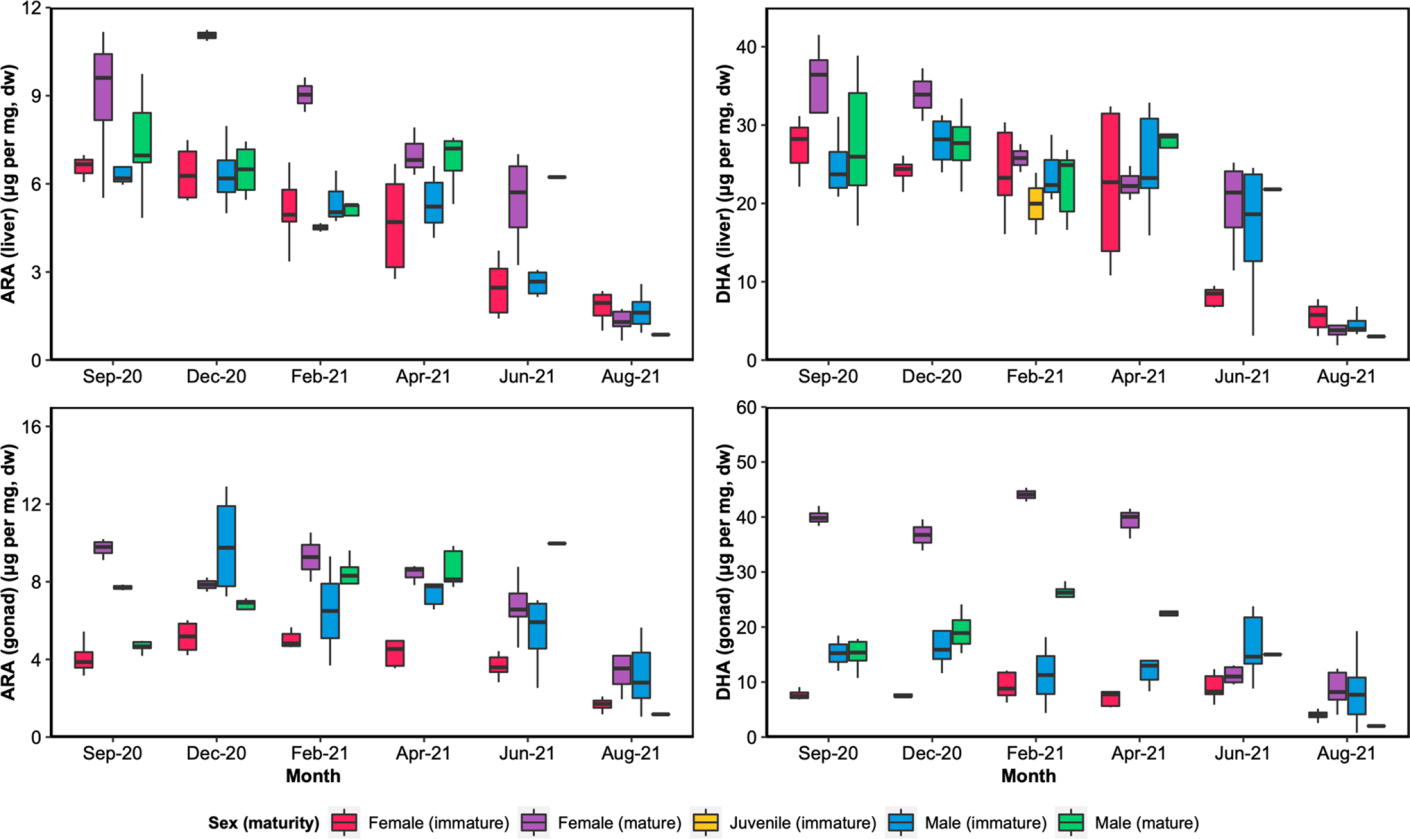
Boxplots representing the concentrations (μg per mg of tissue, dry weight) of ARA (left plots) and DHA (right plots) in the liver (top) and gonad (bottom) of perch sampled in Lake Pääjärvi between September 2020 and August 2021 (*N = *2–8). Juvenile individuals were only collected in February 2021, and their gonad tissue was not sampled

All individuals taken together, the content of ARA and DHA in perch liver followed a similar trend: it was higher in September and gradually decreased until August of the following year when it was the lowest (Fig. 2). In the gonadal tissue, the DHA content in mature females was high compared to other individuals, reaching ~ 40 μg per mg in September and remaining high until the spawning period, after which it dropped and stayed low in June and August (~ 10 μg per mg, Fig. 2). Mature males tended to accumulate DHA in their gonads, however, DHA increased gradually from September reaching lower contents (~ 25 μg per mg in February and April) before the spawning period compared to mature females (Fig. 2).

The DHA content in the gonad for mature females was positively correlated (*p* ≤ 0.05, *r* = 0.66) with their *fasd2* expression in the liver. The DHA content of muscles correlated positively with the liver *fads2* expression for mature males and immature females (*p* ≤ 0.05, *r* = 0.45 and 0.56, respectively), but not for immature males and mature females. Finally, the liver ARA content was negatively correlated (*p* ≤ 0.05, *r* = -0.50) with *fads2* expression for mature males but not for females or immature males.

### Polyunsaturated fatty acids stable isotope values and *fads2* gene expression

**Fig. 3:**
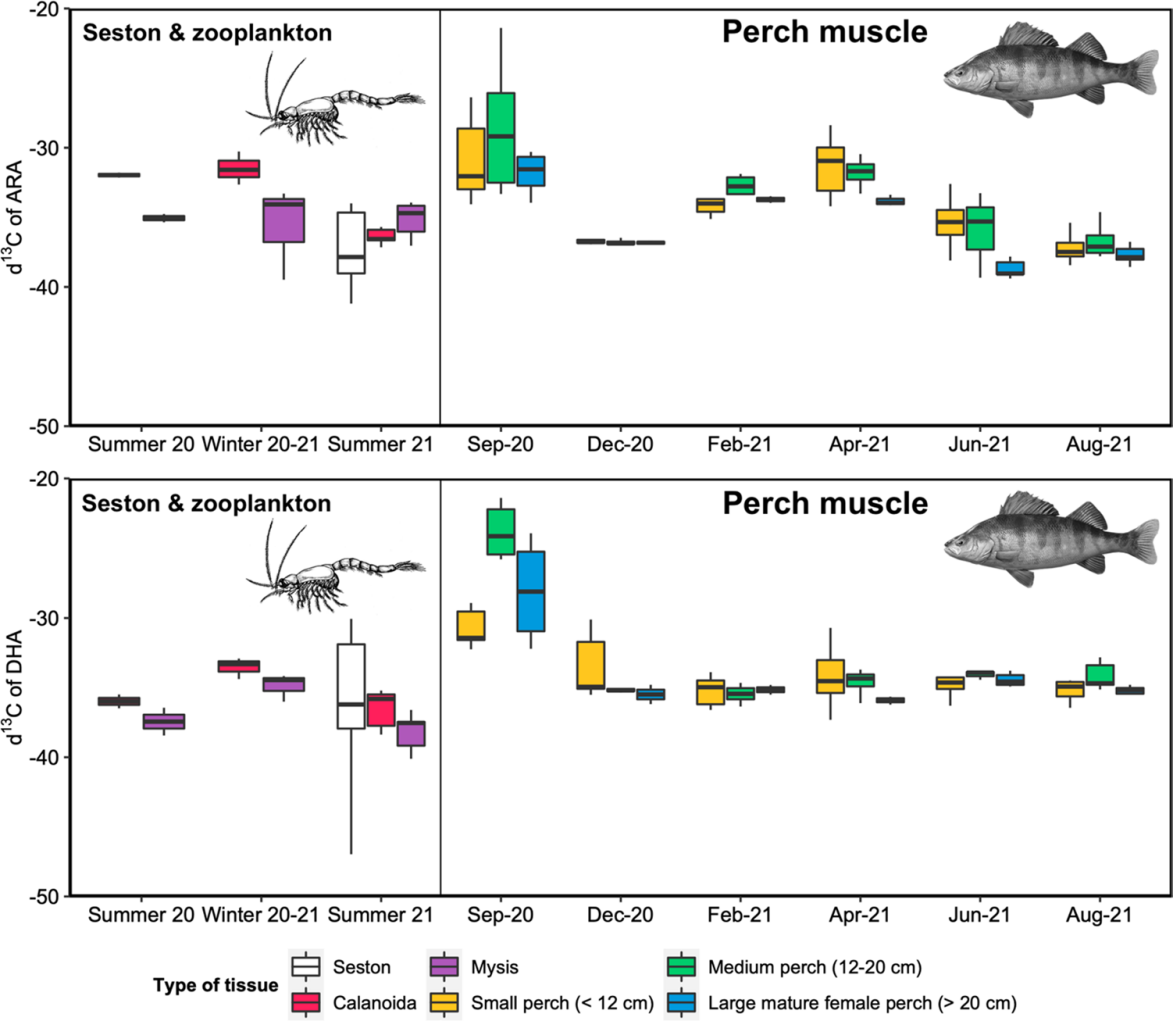
Temporal trends of the δ^13^C values of ARA (top) and DHA (bottom) in the seston, in the main zooplankton prey items of the perch (Calanoida and *Mysis relicta*) and in the perch muscle tissue. Perch were separated by size class in the case of small (< 12 cm) and medium (12–20 cm) sized individuals, but for the large individual (> 20 cm) the emphasis was put on mature females, excluding large males (see main text for explanations). *N = *2–10

The δ^13^C values of DHA (δ^13^C_DHA_) in the muscle were similar all year long and followed those of the prey items, except for September 2020, when values were higher (Fig. 3). The δ^13^C_ARA_values in the perch muscle fluctuated more compared to those of DHA but overall remained within the maximum and minimum values measured in the prey items (Fig. 3).

**Fig. 4:**
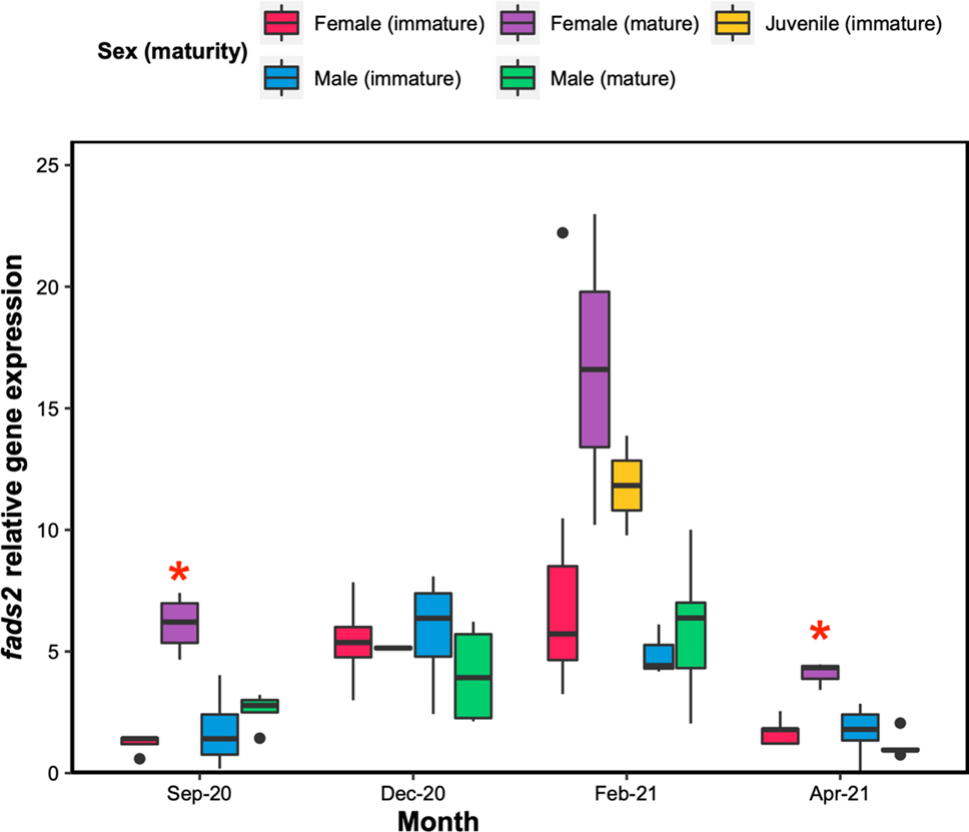
Boxplots representing the relative expression of *fads2* in the liver of perch sampled in Lake Pääjärvi between September 2020 and April 2021 (*N = *2–8). Juvenile individuals were only collected in February 2021. Red asterisk indicates values that are significantly different from all other groups (except between mature females and males for September, *p* = 0.06), for a given month (KW, *p* ≤ 0.05)

Two clear trends were observed regarding the *fads2* gene expression in the liver. First, a seasonal trend: all individuals together, the expression of *fads2* was higher (KW, *p* ≤ 0.05) during the winter months (December and February) compared to September and April (Fig. 4). Second, when comparing individuals based on their sex and maturity stages on a monthly basis, it appeared that mature females had a higher *fads2* expression than any other individuals, except for December (Fig. 4). This was statistically significant in September and April (KW, *p* ≤ 0.05), but not in February.

**Fig. 5:**
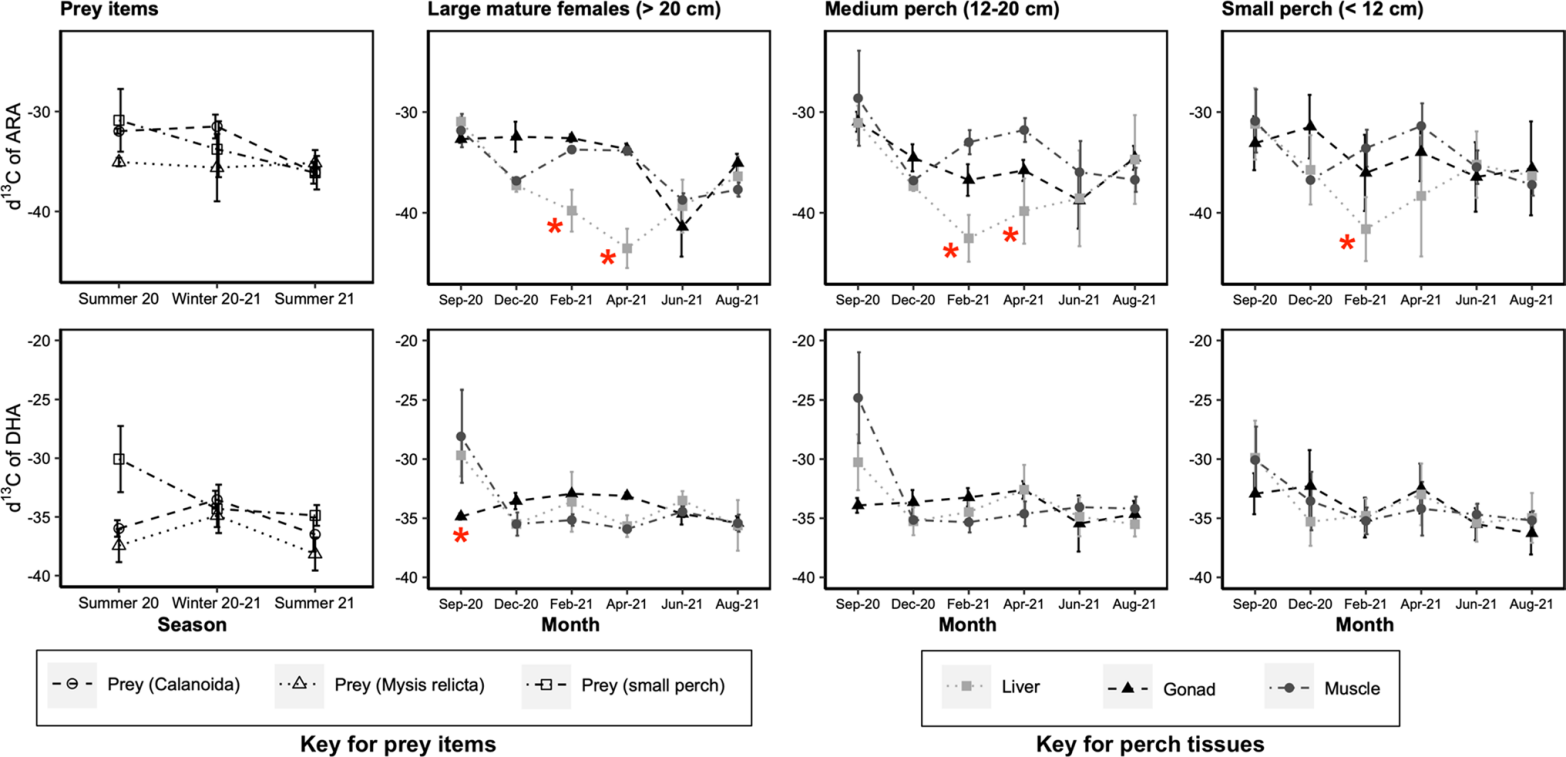
Temporal trends of the δ^13^C values of ARA (top) and DHA (bottom) in the perch tissues (liver, muscle and gonad) and the main potential perch prey items (Calanoida, *Mysis relicta* and small perch muscle). Perch were separated by size class in the case of small (< 12 cm) and medium (12–20 cm) sized individuals, but for the large individual (> 20 cm) the focus was put on mature females, excluding large males (see main text for explanations). Red asterisk indicates values for perch tissues that are significantly different from other tissues and/or from potential preys (KW, *p* ≤ 0.05). *N = *2–10

The δ^13^C_DHA_ values were lower in the gonads of the large mature females in September compared to other tissues or their prey of choice, small perch (KW, *p* ≤ 0.05, Fig. 5). The δ^13^C_ARA_ values in the liver of all individuals tended to decline during the cold months (February and April) compared to other tissues or prey items (Fig. 5). This was particularly noticeable in the large mature females, but also significant in other fish size categories.

## Discussion

Mature female perch in the boreal Lake Pääjärvi seem to take advantage of the short but productive summer window to prepare for the next spring reproduction already during late summer, and early autumn. This behavior is commonly observed in terrestrial animals that reproduce during winter (e.g., bears), and often characterized by hyperphagia (pre-winter fattening), but also by physiological mechanisms favorizing lipid storage before winter, and lipid rather than protein depletion during hibernation (Hissa et al. 1998; Mustonen and Nieminen 2018; Shimozuru et al. 2016; Thiemann et al. 2006). Perch had a noticeably higher GSI already in September compared to August, although they were not consecutive months in our sampling. Moreover, the DHA content measured in their gonads was already at its maximum (~ 40 μg per mg) in September and remained steady until spawning. The onset of gonadal development likely occurred after the summer period, between August and September, when the water temperature dropped, and was characterized by the early accumulation of yolk vesicles, as reported previously for perch (Kirczuk et al. 2015; Sulistyo et al. 1998). Although not measured for the present study, the water temperature dropped noticeably (from 20.5 to 13.6 °C) between August and September 2020 in Lake Pääjärvi (Laiho 2022).

While the *fads2* gene in the perch has not been fully functionally characterized yet (i.e., only for ∆6 desaturation activity towards C18 precursors), there is experimental evidence suggesting possible endogenous DHA biosynthesis by perch (Geay et al. 2015). Two alternative perch *fads2* splicing transcripts have yet to be functionally characterized and, as observed in other fish species, they may be involved in the other desaturation steps needed for the biosynthesis of ARA, EPA and/or DHA (Geay et al. 2016; Matsushita et al. 2023). To our knowledge, the present study is the first to explore the seasonal variability in the expression of *fads2* in wild fish. Individual differences were characterized by the large mature females having generally higher *fads2* expression compared to males or immature females. The increase in GSI and the accumulation of DHA into the gonads coupled with the high *fads2* expression in the liver of mature females in September suggest that at least some of this DHA might be actively synthesized as part of the reproductive investment, as already mentioned before in the literature (Rudchenko and Yablokov 2018), assuming that perch *fads2* is able to perform the required desaturation steps. This is supported by the lower δ^13^C_DHA_ values of the gonads in September compared to other tissues or prey: because enzymatic processes usually use ^12^C faster than ^13^C (Twining et al. 2020), lower δ^13^C values can be expected when a molecule is synthesized rather than taken from the diet or mobilized from other tissues. This could be a mechanism for the perch to secure some spawning resources even in the case of a very harsh winter. In fish, an appropriate supply of DHA to the developing embryos is primordial, as maternal deficiency has been shown to negatively and irreversibly affect larval fitness (e.g., reduced antipredator escape behavior) (Fuiman and Ojanguren 2011).

Strong female-biased sexual dimorphism occurs in perch populations (Estlander et al. 2017), where mature females grow up to larger sizes than mature males and develop much larger gonads, as observed in the present study. A higher *fads2* expression in the tissues of female fish compared to males has already been reported before for some species. For example, higher *fads2* copy numbers in females compared to males were reported in stickleback (*Gasterosteus* sp.) populations and explained by the linkage of *fads2* to the X chromosome (Ishikawa et al. 2019). Female sea bream (*Sparus aurata*) individuals were shown to have a higher *fads2* gene expression in blood cells compared to males (Ferosekhan et al. 2020; Turkmen et al. 2019). This observation is not specific to fish, as similar findings were reported in female humans and rats (Burdge et al. 2017; Kitson et al. 2012). In fish, these authors suggested that higher DHA requirements for females, and more specifically for later embryogenesis, was one possible explanation for this difference with males. Our observations for the mature female perch of Lake Pääjärvi fit that narrative quite well, as their DHA content in the gonad (but not in other tissues) was significantly and positively correlated with *fads2* expression in the liver. On the other hand, mature males had their highest GSI in September, in accordance with previous findings (Sulistyo et al. 2000). However, this was not associated with higher DHA content in their gonads until later during the reproduction cycle (February). This contrasts with the early DHA accumulation observed in the mature female. Mature males showed similar *fads2* liver expression levels compared to immature males, suggesting that mature males do not actively accumulate synthesized DHA into their gonads as much as females and thus likely use their biosynthesis capabilities differently.

After a large initial DHA investment in the gonad during late summer, mature females still need to incorporate DHA in their gonads during the following months, as the gonad size keeps increasing while its DHA content remains constant (~ 40 μg per mg) until spawning. However, even though *fads2* expression in the liver of mature females appeared to remain higher than for other individuals during the months following September (except in December), there was no isotopic evidence that synthesized DHA was further incorporated into the gonads, as no significant differences in δ^13^C_DHA_ values were observed between gonads and other tissues or prey items. With decreasing temperatures and light conditions, foraging activity decreased from September onwards, as illustrated by the overall lower stomach fullness index between September and April compared to June and August. During the summer period, when primary production is high and when food is more easily available, perch need to recover from the demanding spawning period first. It is then possible that the dietary DHA alone is not enough to cover the cost of the high gonadal DHA requirements of mature females and/or that the dietary DHA is (at least partly) stored elsewhere for other purposes (such as lipids reserves), forcing mature females to synthesize a large part of the DHA destined to the gonad, when possible (i.e., when DHA precursors are abundant in the diet). Indeed, perch store most of their dietary fat in their perivisceral fat, including DHA, rather than in their muscle tissue (Blanchard et al. 2005; Xu et al. 2001). It is plausible that some of these stored DHA reserves are later incorporated into the gonads, during the winter period (Murray et al. 2015). Unfortunately, this cannot be confirmed here as the perivisceral fat was not sampled or monitored in the present study.

We also observed seasonal differences in the liver expression of *fads2* in the perch. The overall expression of *fads2* (all individuals taken together) was higher during the winter months: this was especially noticeable in February, which was the coldest sampling month. The fact that the expression of *fads2* increases during the winter months even for the immature individuals strongly implies that *fads2* has important physiological implications other than reproduction for the perch of Lake Pääjärvi, such as over-wintering. In winter, light conditions are poor due to the shorter days but also due to the ice and snow cover, decreasing the visual light penetration to at least half (Piro et al. 2023), making it difficult for perch to see and capture prey (Bergman 1988). Primary production is also much decreased compared to summer, which limits LC-PUFA supply for consumers. Consequently, the cold winter months are characterized by low activity, lowered prey capture ability, and thus starvation for perch (Laiho 2022). Nonetheless, having proper DHA supply or reserves is even more important during that period, as DHA is key to the maintenance of cell membrane fluidity in cold conditions (Arts and Kohler 2009), but also to processes such as visual acuity (Pilecky et al. 2021), which is critical when the ice and snow cover reduces light penetration in the lake. Accordingly, in our dataset, the DHA content in the muscle was at its highest in February. The increased *fads2* expression in the liver during the cold months, and especially its peak in February when the temperature was at its lowest in our sampling, suggests that the perch may not be able to get all the required DHA from its diet or reserves during that period, but may be able to compensate for it by synthesizing some to provide for its needs. However, this assumption cannot be verified here as diet consumption was low at that time and perivisceral fat tissues were not sampled. Future studies may target DHA requirements of other vital organs, such as brain or eyes, as the brain of perch was the second most important tissue in terms of *fads2* expression (Geay et al. 2016).

In addition to DHA, ARA is also a product of *fads2*; however, its role in fish physiology has been rather poorly investigated in contrast with EPA and DHA (Xu et al. 2022). Perch displayed a clear contrast regarding their ARA content in the muscle, with values being higher during the colder months compared to summer. The δ^13^C_ARA_ values of the liver were noticeably lower, suggesting bioconversion, than in other tissues in February and April. This trend was particularly noticeable in the large mature females and may suggest that some of this ARA was actively synthesized by *fads2* during the winter months, assuming that perch *fads2* is capable of desaturation activity towards C20 precursors. However, it must be noted that the δ^13^C_LA_ values were also lower in the muscle of all individuals compared to other tissues or prey items, making the interpretation of the data for LA and ARA complicated. Retro-conversion of DPA (22:5ω6, docosapentaenoic acid) into ARA has been shown as an important alternative synthesis pathway for ARA in the Crustacea *Daphnia magna* (Strandberg et al. 2014). Similarly, retro-conversion of n-6 C20-PUFA to LA has been shown to occur in mammals (Lin and Salem 2005). While we could not find evidence of such processes in fish in the literature, their existence cannot be excluded. Although most enzymes involved in the biosynthesis of PUFA have a greater affinity for n-3 PUFA (Bell and Tocher 2009), there is a constant competition between n-3 and n-6 PUFA for these enzymes (Ahlgren et al. 2009), possibly suggesting ARA being synthesized as a by-product alongside DHA.

The C20-PUFA ARA and EPA are the precursors of eicosanoids. ARA-derived eicosanoids promote inflammation, while those from EPA have been described as anti-inflammatory (Arts and Kohler 2009). ARA-derived eicosanoids have also been shown as important regarding stress resistance (Xu et al. 2022). A recent study conducted with striped bass (*Morone saxatilis*) suggested that ARA might be important in stress conditions involving sub-optimal temperatures, as the pro-inflammatory eicosanoids derived from ARA are crucial when fish are exposed to low temperature for a long period (Araújo et al. 2021). This could potentially explain why our perch appeared to increase its ARA content in the muscle as early as September (marked by a temperature drop after the summer) and then maintained it at these levels during the winter. In terrestrial systems, mosses (Bryophyta) are known to be rich in LC-PUFA, especially ARA, compared to other plants (Beike et al. 2014). While they aren’t generally grazed by vertebrates, mosses are actively consumed by some species in cold environments (Soininen et al. 2017), which suggests that ARA is also important for cold resistance in terrestrial animals (Prins 1982).

ARA is also important in fish reproduction. For perch, an in vitro study showed that ARA (but not the omega-3 EPA or DHA) was able to induce the production of 17,20*β*-dihydroxy-4-pregnen-3-one (DHP), the hormone produced by vitellogenic follicles undergoing final meiotic maturation (Henrotte et al. 2011). Similar importance of ARA in regulating reproduction has been described for mammals as well (Zhang et al. 2019). In the brown bear (*Ursus arctos*), where cubs are born during the winter, the proportion of DHA and ARA in the plasma of adults is at its maximum in winter (Hissa et al. 1998). Migration to warmer waters, which are rich in n-6 LC-PUFA, has been hypothesized as key to the acquisition of ARA for reproduction in a marine mammal that evolved from a terrestrial mammal, the grey whale (*Eschrichtius robustus*) (Caraveo-Patiño et al. 2009).

In conclusion, the present study suggests that perch can modulate its biosynthesis of physiologically required PUFA during starvation or at cold temperatures, typical of over-wintering conditions, or during high energetic demands (gonadal development). As observed with other species, mature female perch appeared to have a higher capability for the synthesis of these physiologically required PUFA compared to other individuals. This is likely explained by their high requirement of DHA storage in the gonads for later successful embryogenesis. However, more research is needed to fully functionally characterize perch *fads2* (more precisely its alternative splicing transcripts) and confirm these findings. The long winter season in boreal lakes is characterized by low temperatures, low light conditions, reduced foraging activity, and thus long fasting periods for perch. Nonetheless, the *fads2* expression was at its highest in winter, including both sex and all size categories, suggesting that the biosynthesis of physiologically required PUFA, such as DHA or ARA, might be needed to cope with these environmental and physiological stressful conditions. The inclusion of other tissues (perivisceral fat, skeleton, brain, and eye) in future studies could help better understand the seasonal dynamics of PUFA in perch or other fish capable of synthesizing these LC-PUFA. Studying *fads2* in fish inhabiting different lake ecosystems would increase our understanding how other fish intrinsically respond to the paucity of dietary PUFA to optimize their fitness. Terrestrial animals also do possess the desaturase genomic architecture required to convert LA and ALA to LC-PUFA, however, the related literature is largely biased towards laboratory-raised, domesticated or hatchery-reared species (Castro et al. 2012; Twining et al. 2016). Like for fish, there is little to no information regarding the role or dynamics of desaturase genes in these processes for wild animals in terrestrial ecosystems, thus more research is needed.

### Supplementary Information

Below is the link to the electronic supplementary material.

Supplementary file1 (DOCX 463 KB)

## Data Availability

All data collected and used during the present study are publicly available at https://doi.org/10.17011/jyx/dataset/83987.
