# Supplementary material for: Preparing for the future offspring: European perch (Perca fluviatilis) biosynthesis of physiologically required fatty acids for the gonads happens already in the autumn
Source: Oecologia. 2023 Nov 17;203(3-4):477–89. doi: 10.1007/s00442-023-05480-0 (PMC10684423; doi:10.1007/s00442-023-05480-0)
Supplement: Supplementary file 1 — Supplementary file1 (DOCX 463 KB) [file 442_2023_5480_MOESM1_ESM.docx]

**Title**

Preparing for the future offspring – European perch (*Perca fluviatilis*) biosynthesis of physiologically required fatty acids for the gonads happens already in the autumn

**Authors**

Cyril Rigaud ^a,*^, Kimmo K. Kahilainen ^b^, Marco L. Calderini ^a^, Matthias Pilecky ^c,d^, Martin J. Kainz ^c,d^, Marja Tiirola ^a^, Sami J. Taipale ^a^

**Affiliations**

^a^ Department of Biological and Environmental Sciences, University of Jyväskylä, Jyväskylä, Finland

^b^ Lammi Biological Station, University of Helsinki, Lammi, Finland

^c^ WasserCluster Lunz – Inter-university Center for Aquatic Ecosystem Studies, Lunz am See, Austria

^d^ Danube University Krems, Research Lab of Aquatic Ecosystem Research and -Health, Krems, Austria

* Address correspondence to [cyril.c.rigaud@jyu.fi](mailto:cyril.c.rigaud@jyu.fi)

**Supplementary materials**

**Measurement of the *fads2* gene expression in the liver**

RNA was extracted from liver samples using a Chemagic™ 360 and the Chemagic™ Viral DNA/RNA 300 Kit H96 following the manufacturer’s instructions (PerkinElmer, Waltham, MA, USA). The RNA was treated with DNAse and reverse transcribed to cDNA using the Maxima First Strand cDNA Synthesis Kit (Thermo Fisher Scientific, Waltham, MA, USA). The effectiveness of the DNase treatment was validated by including randomly chosen negative RT samples. cDNA samples were stored at -20°C.

The degenerate primer sequences for *fads2* and quantitative real-time PCR analyses (qPCR) primer sequences for *fads2*, *ef1-α*, and *β-actin* were all obtained from the literature (Geay et al., 2016). However, as efficiency values for the qPCR primers were not reported, we measured them: it was equal to 94.4%, 96.9%, and 94.2% for *fads2*, *ef1-α*, and *β-actin*, respectively. All amplification reactions were done in a volume of 25 μL containing 2 μL of cDNA, 0.75 μL (each) of the forward and reverse primers (working solution at 10 μM), 9 μL of sterile water and 12.5 μL of 2X Maxima SYBR Green/Fluorescein qPCR Master Mix (Thermo Fisher Scientific). The reactions were run on a CFX96 Real-Time PCR cycler (Bio-Rad, Hercules, CA, USA). The protocol was 10 min at 95°C, 40 cycles of 15 s at 95°C, 30 s at 60°C, and 30 s at 72°C, and ended with 10 s at 95°C followed by melting curves from 65°C to 95°C. A single melting temperature peak was observed in the dissociation curves for each pair of primers. No amplification was observed in no-template controls (sterile water instead of cDNA) and negative RT samples.

Before qPCR analyses, the presence of the *fads2* gene in the liver of the perch from Lake Pääjärvi was verified by using the degenerate primers (Geay et al., 2016). A single product of 739 bp (compared to 738 bp reported by Geay et al. (2016)) was obtained, purified, and sequenced (Eurofins Scientific, Cologne, Germany). The output obtained from the forward and reverse primer sequencing shared a 96.84% and 96.75% similarity, respectively, compared to the sequence published by Geay et al. (2016). Most importantly, the qPCR primers for *fads2* perfectly matched the output of our sequencing data. For each sample, the *fads2* gene expression (efficiency corrected) was calculated using the CFX Maestro™ software (Bio-Rad) according to the methods described by Pfaffl (2001) and Vandesompele (2002). The stability of the two reference genes *ef1-α* and *β-actin* was acceptable in the conditions of our study (coefficient of variation below 5%).

**Fatty acids content and stable isotopes analysis**

Total lipids were extracted from seston (filtered samples), zooplankton, and fish tissues using the protocol described by Folch et al. (1957) using chloroform:methanol:water at a 8:4:3 ratio. After extraction, toluene and sulfuric acid were used for the transesterification of fatty acid methyl esters (FAME) at 50°C for 10 h. FAME samples were analyzed with a gas chromatograph (Shimadzu, Kyoto, Japan) equipped with a mass detector (GC-MS) and using helium as a carrier gas and an Agilent (Santa Clara, CA, USA) DB-23 column (60 m × 0.25 mm × 0.15 µm). The column temperature was raised from the initial temperature, 60°C, to 130°C at the rate of 30°C min^−1^, after which the temperature was raised to 170°C at the rate of 7°C min^−1^. Finally, the temperature was raised to 220°C at the rate of 1.5°C min^−1^ and held at that temperature for 10 minutes. The injection temperature was 260°C, and the interface temperature was 250°C. Total gas flow in the column was 2.0 mL min^−1^ and linear velocity 36.3 cm s^−1^. The identification and quantification followed the previously published method (Taipale et al., 2016). The fatty acid FAME (Larodan AB, Solna, Sweden) was used as the internal standard for the correction of sample FA concentrations.

The δ^13^C values of FA were determined using a GC-MSD (Agilent 7890B GC, Agilent 5977B MSD) connected to an Isotope Ratio Mass Spectrometer (Isoprime precisION, Elementar, Langenselbold, Germany) via combustion interface (Elementar GC5 combustion interface) at the University of Jyväskylä (Finland). Fatty acids were separated using a 30 m ZB-23 column (0.25 mm x 0.15 mm; Phenomenex, Torrance, CA, USA) and then oxidized to carbon dioxide in an oxidation reactor at a temperature of 940°C with the reduction reactor kept at 630°C. The temperature program of the GC column started at 50°C and was kept for 1 minute at that temperature, after which the temperature was raised by 10°C min^-1^ to 130°C, then by 7°C min^-1^ to 180°C, and then by 1°C min^-1^ to 210°C and held on that temperature for 3 min. Finally, the temperature was raised to 260°C at the rate of 10°C min^−1^. The total run time was 48.03 minutes. The injector temperature was kept at 280°C. The samples were run against an internal standard, FAME, which was used for drift and linear correction. The δ^13^C value of FAME (internal standard) and methanol (derivatization reagent) were run with EA-SIRMS system (Finnigan DELTAplus Advantage, Thermo Fisher Scientific), which were used to calculate the δ^13^C value of individual FA. The calculated precision for standard FAME was ± 0.4‰, and the accuracy was ± 0.3‰. The δ^13^C values of individual FAME were manually calculated using individual background values. The δ^13^C values of FA were calculated from the δ^13^C value of FAME by correcting the methyl group.

**References**

Folch, J., Lees, M., & Sloane Stanley, G. H. (1957). A simple method for the isolation and purification of total lipides from animal tissues. The Journal of Biological Chemistry, 226(1), 497–509. https://doi.org/10.1016/S0021-9258(18)64849-5

Geay, F., Tinti, E., Mellery, J., Michaux, C., Larondelle, Y., Perpète, E., & Kestemont, P. (2016). Cloning and functional characterization of Δ6 fatty acid desaturase (FADS2) in Eurasian perch (*Perca fluviatilis*). Comparative Biochemistry and Physiology Part B: Biochemistry and Molecular Biology. https://doi.org/https://doi.org/10.1016/j.cbpb.2015.10.004

Pfaffl, M. W. (2001). A new mathematical model for relative quantification in real-time RT–PCR. Nucleic Acids Research, 29(9), e45. https://doi.org/https://doi.org/10.1093/nar/29.9.e45

Taipale, S. J., Vuorio, K., Strandberg, U., Kahilainen, K. K., Järvinen, M., Hiltunen, M., Peltomaa, E., & Kankaala, P. (2016). Lake eutrophication and brownification downgrade availability and transfer of essential fatty acids for human consumption. Environment International, 96, 156–166. https://doi.org/10.1016/J.ENVINT.2016.08.018

Vandesompele, J., de Preter, K., Pattyn, F., Poppe, B., van Roy, N., de Paepe, A., & Speleman, F. (2002). Accurate normalization of real-time quantitative RT-PCR data by geometric averaging of multiple internal control genes. In Genome biology (Vol. 3, Issue 7, p. research0034. 1). BioMed Central.

**Table S1** Physical and chemical parameters measured in Lake Pääjärvi between September 2020 and August 2021

| Variable | September 2020 | December 2020 | February 2021 | April 2021 | June 2021 | August 2021 |
| --- | --- | --- | --- | --- | --- | --- |
| Temperature °C (0-3 m) | 13.6 | 4.2 | 0.8 | 3.1 | 14.9 | 18.8 |
| Compensation depth (m) | 3 | 2.5 | 1.5 | 2.5 | 3 | 3 |
| Oxygen saturation % (0-3 m) | 94.2 | 93.3 | 91.1 | 91.4 | 98.2 | 100.4 |
| Oxygen 0-3 m (mg/L) | 9.8 | 12.2 | 13 | 12.3 | 10 | 9.4 |
| Total phosphorus (μg/L) | 8 | 10 | 10 | 10 | 13 | 7 |
| Total nitrogen (μg/L) | 1278 | 1420 | 1318 | 1537 | 1628 | 1447 |
| Total organic carbon (mg/L) | 11.5 | 11.9 | 12.4 | 12.1 | 13.1 | 12.3 |
| Color (Pt/L) | 73 | 81 | 85 | 87 | 96 | 79 |
| pH | 7.3 | 7.2 | 7.2 | 7 | 7.2 | 7.3 |
| chlorophyll-a (μg/L) | 3.4 | 0.6 | 0.3 | 0.5 | 7.7 | 4.5 |
| Ice thickness (cm) | 0 | 0 | 27 | 0 | 0 | 0 |
| Snow depth (cm) | 0 | 0 | 8.2 | 0 | 0 | 0 |

| Sex (maturity) | Sampling month | N | Total length (cm) | | | Mass (g) | | | Condition factor (CF) | | | Gonadosomatic index | | | | Stomach fullness | | | | Empty stomach (%) | |
| --- | --- | --- | --- | --- | --- | --- | --- | --- | --- | --- | --- | --- | --- | --- | --- | --- | --- | --- | --- | --- | --- |
| Females (immature) | September 2020 | 4 | 11.0 | ± | 0.6 | 11.6 | ± | 1.8 | 0.87 | ± | 0.03 | NA | ± | NA | 0.00 | | ± | 0.00 | 100.0 | |  |
|  | December 2020 | 4 | 14.3 | ± | 3.9 | 34.5 | ± | 26.0 | 0.91 | ± | 0.09 | 0.39 | ± | 0.09 | 5.75 | | ± | 2.28 | 0.0 | |  |
|  | February 2021 | 8 | 15.2 | ± | 5.1 | 48.2 | ± | 42.8 | 0.92 | ± | 0.15 | 0.33 | ± | 0.12 | 2.14 | | ± | 1.64 | 25.0 | |  |
|  | April 2021 | 5 | 16.7 | ± | 5.6 | 60.9 | ± | 57.6 | 0.89 | ± | 0.10 | 0.37 | ± | 0.08 | 0.60 | | ± | 1.20 | 80.0 | |  |
|  | June 2021 | 7 | 13.5 | ± | 3.2 | 30.1 | ± | 23.7 | 1.01 | ± | 0.09 | 0.45 | ± | 0.06 | 5.29 | | ± | 1.16 | 0.0 | |  |
|  | August 2021 | 6 | 13.6 | ± | 3.4 | 31.3 | ± | 22.5 | 1.01 | ± | 0.11 | 0.34 | ± | 0.12 | 3.50 | | ± | 1.61 | 0.0 | |  |
|  |  |  |  |  |  |  |  |  |  |  |  |  |  |  |  | |  |  |  | |  |
| Males (immature) | September 2020 | 4 | 12.3 | ± | 2.0 | 19.6 | ± | 12.2 | 0.92 | ± | 0.08 | 0.07 | ± | 0.01 | 2.25 | | ± | 3.90 | 75.0 | |  |
|  | December 2020 | 4 | 12.3 | ± | 2.5 | 19.0 | ± | 13.3 | 0.89 | ± | 0.02 | 0.16 | ± | 0.08 | 4.50 | | ± | 1.50 | 0.0 | |  |
|  | February 2021 | 3 | 14.8 | ± | 4.1 | 37.3 | ± | 26.6 | 0.91 | ± | 0.05 | 0.11 | ± | 0.02 | 2.00 | | ± | 1.63 | 33.3 | |  |
|  | April 2021 | 7 | 13.4 | ± | 4.0 | 29.4 | ± | 29.5 | 0.87 | ± | 0.13 | 0.18 | ± | 0.06 | 1.14 | | ± | 1.46 | 57.1 | |  |
|  | June 2021 | 6 | 11.0 | ± | 2.9 | 15.2 | ± | 12.3 | 0.97 | ± | 0.05 | 0.12 | ± | 0.05 | 6.17 | | ± | 2.48 | 0.0 | |  |
|  | August 2021 | 7 | 15.7 | ± | 5.2 | 58.7 | ± | 58.3 | 1.04 | ± | 0.12 | 0.09 | ± | 0.04 | 4.14 | | ± | 2.23 | 14.3 | |  |
|  |  |  |  |  |  |  |  |  |  |  |  |  |  |  |  | |  |  |  | |  |
| Females (mature) | September 2020 | 4 | 24.2 | ± | 1.3 | 170.3 | ± | 40.7 | 1.18 | ± | 0.08 | 2.83 | ± | 0.46 | 1.25 | | ± | 1.64 | 50.0 | |  |
|  | December 2020 | 2 | 27.1 | ± | 3.5 | 263.7 | ± | 106.9 | 1.25 | ± | 0.06 | 9.79 | ± | 0.43 | 4.00 | | ± | 0.00 | 0.0 | |  |
|  | February 2021 | 2 | 28.4 | ± | 1.0 | 318.7 | ± | 56.2 | 1.38 | ± | 0.11 | 14.78 | ± | 1.50 | 0.50 | | ± | 0.50 | 50.0 | |  |
|  | April 2021 | 3 | 29.3 | ± | 2.3 | 331.7 | ± | 110.9 | 1.27 | ± | 0.10 | 22.04 | ± | 1.07 | 1.67 | | ± | 1.25 | 33.3 | |  |
|  | June 2021 | 6 | 26.1 | ± | 2.8 | 209.0 | ± | 84.5 | 1.11 | ± | 0.09 | 0.74 | ± | 0.16 | 3.17 | | ± | 2.27 | 0.0 | |  |
|  | August 2021 | 6 | 24.7 | ± | 4.1 | 210.3 | ± | 102.7 | 1.27 | ± | 0.05 | 0.57 | ± | 0.17 | 7.17 | | ± | 2.91 | 0.0 | |  |
|  |  |  |  |  |  |  |  |  |  |  |  |  |  |  |  | |  |  |  | |  |
| Males (mature) | September 2020 | 6 | 17.4 | ± | 4.6 | 70.1 | ± | 58.9 | 1.05 | ± | 0.08 | 6.67 | ± | 1.33 | 3.00 | | ± | 3.16 | 50.0 | |  |
|  | December 2020 | 4 | 18.0 | ± | 5.5 | 81.1 | ± | 76.6 | 1.00 | ± | 0.09 | 4.51 | ± | 0.60 | 4.75 | | ± | 2.17 | 0.0 | |  |
|  | February 2021 | 5 | 20.9 | ± | 2.0 | 102.5 | ± | 41.0 | 1.07 | ± | 0.08 | 4.21 | ± | 0.78 | 0.80 | | ± | 0.75 | 40.0 | |  |
|  | April 2021 | 5 | 19.8 | ± | 5.9 | 103.7 | ± | 83.3 | 0.99 | ± | 0.11 | 3.41 | ± | 1.46 | 0.40 | | ± | 0.49 | 60.0 | |  |
|  | June 2021 | 1 | 20.9 | ± | NA | 79.9 | ± | NA | 0.88 | ± | NA | 0.25 | ± | NA | 2.00 | | ± | 0.00 | 0.0 | |  |
|  | August 2021 | 1 | 20.0 | ± | NA | 95.7 | ± | NA | 1.20 | ± | NA | 0.09 | ± | NA | 3.00 | | ± | 0.00 | 0.0 | |  |
|  |  |  |  |  |  |  |  |  |  |  |  |  |  |  |  | |  |  |  | |  |
| Juveniles (immature) | February 2021 | 2 | 7.0 | ± | 0.5 | 2.5 | ± | 0.02 | 0.71 | ± | 0.02 | NA | ± | NA | 1.50 | | ± | 1.50 | 50.0 | |  |

**Table S2** Biotic and diet parameters of the perch captured in Lake Pääjärvi between September 2020 and August 2021. Fulton’s condition factor was used and stomach fullness is estimated using points method with scale 0 (empty) to 10 (maximum full)

**Table S3** Shares (relative percentage) of the stomach content of perch captured in Lake Pääjärvi between September 2020 and August 2021. Perch were split by size classes: large (> 20 cm), medium (12-20 cm) and small (< 12 cm)

| **Size class** | **Prey item** | **Sept-20** | **Dec-20** | **Feb-21** | **Apr-21** | **Jun-21** | **Aug-21** |
| --- | --- | --- | --- | --- | --- | --- | --- |
| Large (> 20 cm) | Calanoida | - | - | - | - | 4.3 | - |
|  | Copepoda | - | - | - | - | 3.6 | - |
|  | Crayfish | - | - | - | - | 2.9 | - |
|  | Fish (Cyprinidae) | - | - | - | - | 21.4 | - |
|  | Fish (Perch, *Perca fluviatilis*) | - | - | 71.4 | - | 19.3 | 63.6 |
|  | Fish (Percidae) | - | - | - | - | - | 9.1 |
|  | Fish (Pikeperch, *Sander lucioperca*) | - | - | - | - | - | 4.5 |
|  | Fish (Roach, *Rutilus rutilus*) | - | - | - | - | 7.1 | - |
|  | Fish (Ruffe, *Gymnocephalus cernua*) | - | 87.5 | - | - | - | - |
|  | Fish (Salmonidae) | - | - | - | - | - | 2.3 |
|  | Fish (unidentified) | 100.0 | - | - | 100.0 | 20.7 | 20.5 |
|  | *Leptodora kindtii* | - | - | - | - | 20.7 | - |
|  | *Mysis relicta* | - | 12.5 | 28.6 | - | - | - |
| Medium (12-20 cm) | *Asellus aquaticus* | 7.4 | - | - | - | - | - |
|  | *Bosmina sp.* | - | - | - | - | 1.4 | - |
|  | Calanoida | - | - | - | - | 7.0 | - |
|  | Chironomidae | 92.6 | - | 4.8 | - | 1.4 | - |
|  | Copepoda | - | - | - | - | 12.7 | - |
|  | *Daphnia* sp. | - | - | - | - | 14.1 | - |
|  | Fish (Perch, *Perca fluviatilis*) | - | - | - | - | - | 100.0 |
|  | Fish (unidentified) | - | - | 28.6 | - | 21.1 | - |
|  | *Gammaracanthus lacustris* | - | - | 19.0 | - | - | - |
|  | *Leptodora kindtii* | - | - | - | - | 21.1 | - |
|  | *Mysis relicta* | - | 100.0 | 47.6 | - | 0.7 | - |
|  | Surface insects | - | - | - | - | 20.4 | - |
| Small (< 12 cm) | *Asellus aquaticus* | - | - | - | 18.2 | - | - |
|  | *Bosmina sp.* | - | - | - | - | 0.7 | - |
|  | Calanoida | - | - | - | 54.5 | 90.0 | 9.1 |
|  | Chironomidae | - | - | - | - | 0.7 | - |
|  | Copepoda | - | 15.6 | - | - | - | - |
|  | Cyclopoida | - | - | - | - | 4.5 | 67.3 |
|  | *Daphnia* sp. | - | - | - | - | 1.6 | 14.5 |
|  | Fish (unidentified) | - | - | - | - | 0.1 | - |
|  | Insects (unidentified) | - | - | - | - | - | 2.7 |
|  | *Leptodora kindtii* | - | - | - | - | 2.2 | 6.4 |
|  | *Mysis relicta* | - | 84.4 | 100.0 | 27.3 | - | - |


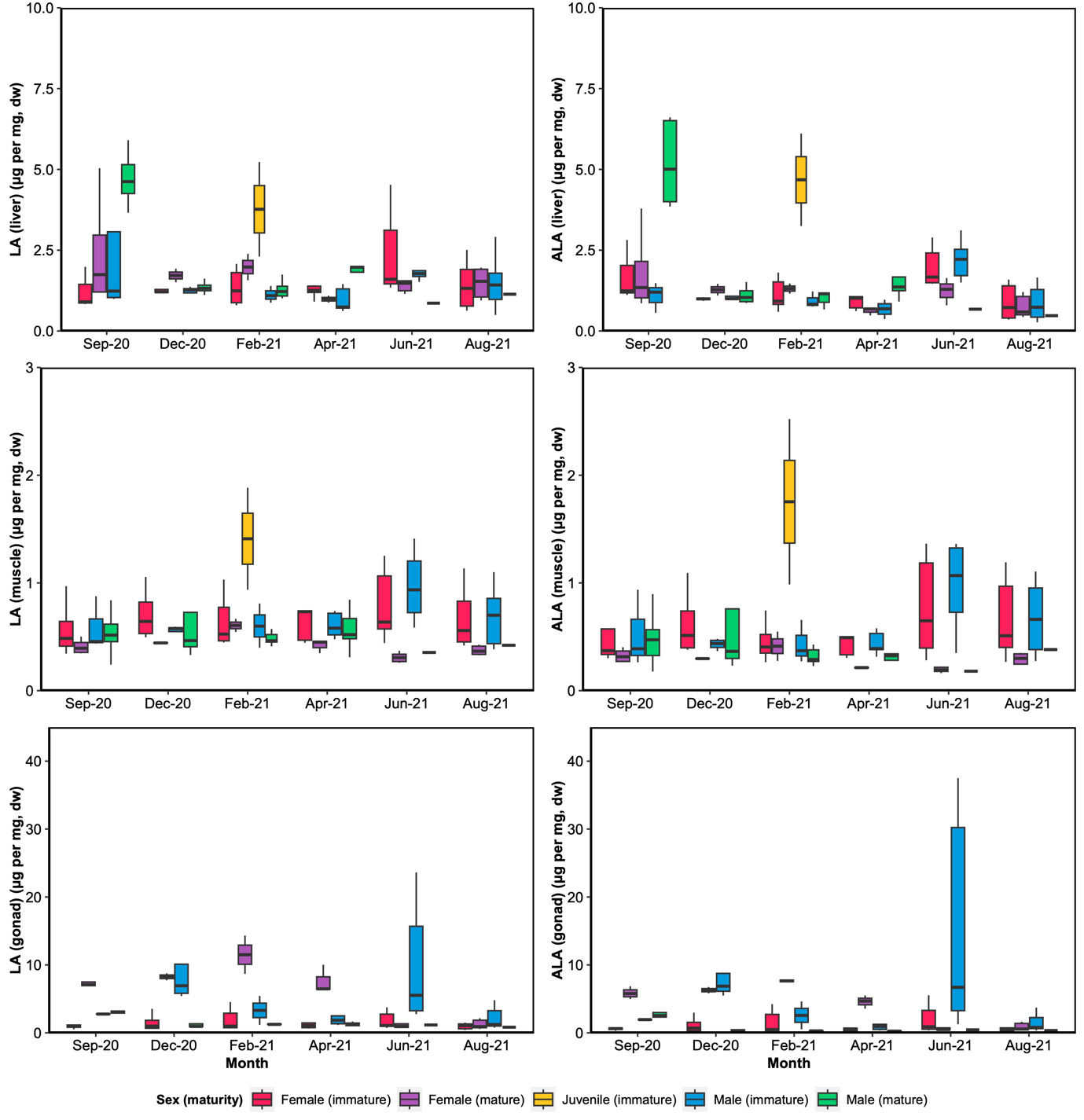


**Fig. S1** Boxplots representing the concentrations (μg per mg of tissue, dry weight) of linoleic acid (LA, left plots) and alpha-linoleic acid (ALA, right plots) in the liver (top), muscle (middle) and gonad (bottom) of perch sampled in Lake Pääjärvi between September 2020 and August 2021 (N = 2-8). Juvenile individuals were only collected in February 2021, and their gonad tissue was not sampled


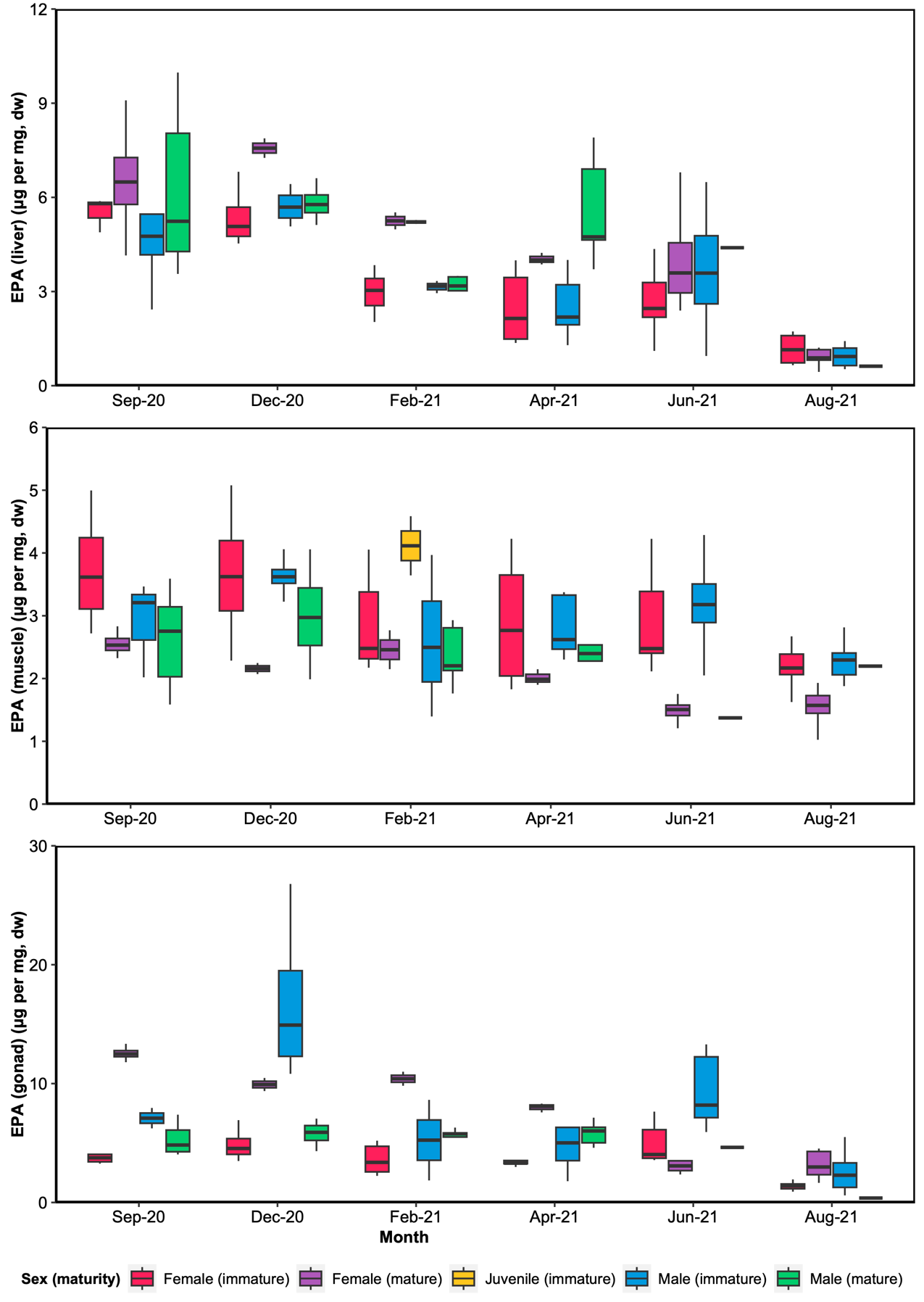


**Fig. S2** Boxplots representing the concentrations (μg per mg of tissue, dry weight) of eicosapentaenoic acid (EPA) in the liver (top), muscle (middle) and gonad (bottom) of perch sampled in Lake Pääjärvi between September 2020 and August 2021 (N = 2-8). Juvenile individuals were only collected in February 2021, and their gonad tissue was not sampled
